# Oxygen extraction fraction is not uniform in human brain: a positron emission tomography study

**DOI:** 10.1186/s12576-023-00880-6

**Published:** 2023-10-12

**Authors:** Hiroshi Ito, Masanobu Ibaraki, Ryo Yamakuni, Motoharu Hakozaki, Naoyuki Ukon, Shiro Ishii, Kenji Fukushima, Hitoshi Kubo, Kazuhiro Takahashi

**Affiliations:** 1 Department of Radiology and Nuclear Medicine, Fukushima Medical University, 1 Hikariga-Oka, Fukushima, 960-1295 Japan; 2 Advanced Clinical Research Center, Fukushima Medical University, Fukushima, Japan; 3 Department of Radiology and Nuclear Medicine, Akita Research Institute of Brain and Blood Vessels, 6-10 Senshu-Kubota-Machi, Akita, 010-0874 Japan; 4 School of Medical Sciences, Fukushima Medical University, Fukushima, Japan

**Keywords:** Brain, CBF, CMRO_2_, OEF, PET

## Abstract

The regional differences in cerebral oxygen extraction fraction (OEF) in brain were investigated using positron emission tomography (PET) in detail with consideration of systemic errors in PET measurement estimated by simulation studies. The cerebral blood flow (CBF), cerebral blood volume (CBV), OEF, and cerebral metabolic rate of oxygen (CMRO_2_) were measured on healthy men by PET with ^15^O-labeled gases. The OEF values in the pons and the parahippocampal gyrus were significantly smaller than in the other brain regions. The OEF value in the lateral side of the occipital cortex was largest among the cerebral cortical regions. Simulation studies have revealed that errors in OEF caused by regional differences in the distribution volume of ^15^O-labeled water, as well as errors in OEF caused by a mixture of gray and white matter, must be negligible. The regional differences in OEF in brain must exist which might be related to physiological meanings.Article title: Kindly check and confirm the edit made in the article title.I have checked the article title and it is OK as is.

*Trial registration*: The UMIN clinical trial number: UMIN000033382, https://www.umin.ac.jp/ctr/index.htm

## Background

Measurement of cerebral blood flow (CBF), cerebral blood volume (CBV), cerebral oxygen extraction fraction (OEF), and cerebral metabolic rate of oxygen (CMRO_2_) by positron emission tomography (PET) with ^15^O-labeled carbon dioxide (C^15^O_2_), ^15^O-labeled carbon monoxide (C^15^O), or ^15^O-labeled oxygen (^15^O_2_) is widely used for investigation into the pathophysiology of occlusive cerebrovascular disease [1–7]. Decreased cerebral perfusion pressure below the lower limit of cerebral autoregulation due to major cerebral arterial occlusive disease causes a decrease in CBF with an increase in OEF, for maintenance of CMRO_2_ (misery perfusion).

OEF, which is used as an indicator of hemodynamic hypoperfusion, corresponds to the ratio of CMRO_2_ to CBF. Normal values of OEF have been reported to be about 0.4 in cerebral cortical regions [8, 9]. There are several reports of normal values of CBF, CBV, OEF, and CMRO_2_ [8–12], and regional differences in CBF, CBV, and vascular mean transit time (MTT) have been investigated in detail with analyses of systemic errors in PET measurement [13]. Regional differences in cerebral vascular response to P_a_CO_2_ changes have also been investigated showing regional differences in cerebral vascular tone in relation with those in MTT [14]. There are some reports about regional differences in CMRO_2_ and OEF [15, 16]; however, such regional differences have not been investigated in detail with analyses of systemic errors based on the quantification theory in PET measurement [17].

To estimate hemodynamic hypoperfusion in occlusive cerebrovascular disease, the regional differences in OEF in healthy subjects should be clarified. In the present study, CBF, CBV, OEF and CMRO_2_ were measured with ^15^O-labeled gases using the integrated design of the positron emission tomography/magnetic resonance imaging (PET/MRI) scanner system, which allows simultaneous data acquisition for PET and MRI [18]. The regional differences in OEF in brain were investigated in detail with consideration of systemic errors in PET measurement estimated by simulation studies [17].

## Methods

### Subjects

Nine healthy men (20–27 years) were recruited who were included in the previous study [12]. No subject had notable organic lesions in the brain according to MRI. The subjects were free of somatic, neurological and psychiatric disorders on the basis of their medical history. They were free from medications having central nervous action. The study was approved by the Institutional Review Board of Fukushima Medical University, Fukushima, Japan, and written informed consent was obtained from all subjects.

### PET/MRI experimental procedure

All PET studies were performed using a Siemens mMR PET/MRI scanner, which provides 127 sections with an axial field of view of 25.8 cm [19]. The intrinsic spatial resolution was 4.3 mm in-plane and 4.3 mm full-width at half maximum (FWHM) axially. Data were acquired in three-dimensional mode, and scatter was corrected [20]. PET measurements with the steady-state method of ^15^O-labeled gases, C^15^O, ^15^O_2_, and C^15^O_2_ were performed on all subjects [9, 21]. PET scanning protocol was according to our previous report [12]. Static PET scanning was started 3 min after 1 min of continuous inhalation of C^15^O gas (a total of approximately 3 GBq supplied by mouth). The duration of the scanning was 4 min. Then, static PET scanning was performed during inhalation of ^15^O_2_ gas after equilibrium had been reached and confirmed by the head radioactivity curve (a total of approximately 8.4 GBq supplied by mouth). The duration of the scanning was 10 min, and the time from the beginning of inhalation to the beginning of scanning was 10 min. Static PET scanning was performed during inhalation of C^15^O_2_ gas using the same protocol as with ^15^O_2_ gas (a total of approximately 2.8 GBq supplied by mouth). During each PET scan, arterial blood sampling was performed at three times to measure the radioactivity concentration in the blood and plasma. Arterial blood gases were also measured. Total oxygen content in arterial blood was estimated from P_a_O_2_, pH and hemoglobin concentration (Hb) [9]. The total volume of blood samples was about 30 mL. All PET measurements were performed without instructions for eye closure.

All MRI studies were performed using a Siemens mMR PET/MRI scanner, equipped with a 3.0-T MR scanner, during and between PET scanning. Dixon sequence (DIXON) [3D-VIBE (volumetric interpolated breath-hold examination), TR: 3.56 ms, TE: 1.23 ms and 2.46 ms; field of view: 500 mm, slice thickness: 3.12 mm, resolution: 2.6 × 2.6 × 3.1 mm, 1 slab: 128 slices] and Dixon sequence with model-based bone segmentation (DIXONbone) were performed for attenuation correction of PET without and with consideration of attenuation by bone, respectively [22, 23]. Three dimensional volumetric T1-weighted images (T1WI) and T2-weighted images (T2WI), diffusion-tensor images, arterial spin labelling images, and MR angiography were also acquired. In the present study, T1WI (three-dimensional MPRAGE magnetization prepared rapid acquisition with gradient echo sequence, TR: 1800 ms, TE: 1.98 ms, flip angle 9°; field of view: 250 mm, acquisition matrix size: 256 × 256, slice thickness: 1.0 mm) were used for analyses.

Image reconstruction of PET was carried out by the ordered-subset expectation maximization (OSEM) algorithm (iterations: 3, subsets: 21) with postreconstruction Gaussian filter of 5 mm FWHM. Two attenuation corrections for PET using DIXON and DIXONbone were applied.

### Calculation of parametric images

Using reconstructed PET images, the radioactivity concentrations in arterial blood and plasma, and arterial blood gases data, the parametric images of CBF, CBV, OEF and CMRO_2_ were calculated [21]. In measurement of OEF and CMRO_2_, a correction for the presence of intravascular ^15^O_2_ was applied [17, 24]. The parametric images were calculated for attenuation correction by DIXON and DIXONbone.

### Data analysis

MR images (T1WI) were transformed into standard brain size and shape by linear and nonlinear parameters using SPM8 (anatomic standardization) [25, 26]. All PET images were also transformed into standard brain size and shape using the same parameters as the MR images. Thus, the brain images of all subjects had the same anatomic format. Gray matter, white matter, and cerebrospinal fluid images were segmented and extracted from all anatomically standardized MR images by applying voxel-based morphometry methods with the SPM8 system [27, 28]. These segmented MR images indicate the tissue fraction of gray or white matter per voxel (mL/mL). All anatomically standardized PET, gray matter and white matter images were smoothed with a 5-mm FWHM isotropic Gaussian kernel, because final spatial resolution of the PET camera was approximately 5 mm FWHM.

Regions of interest (ROIs) were drawn on all anatomically standardized PET images and T1WI. Circular ROIs (10 mm in diameter) were defined for the cerebellar cortex, pons, thalamus, putamen, base side of the frontal cortex, convexity side of the frontal cortex, parahippocampal gyrus, lateral side of the temporal cortex, cuneus of the occipital cortex, lateral side of the occipital cortex, inferior parietal lobule cortex, and centrum semiovale. Values in each brain region were compared using paired *t* test (*P* < 0.05) adjusted for multiple comparisons with Bonferroni correction.

### Error analysis

#### *Errors in measurement of OEF caused by regional differences in the distribution volume of *^*15*^*O-labeled water*

The distribution volume of ^15^O-labeled water is assumed to be a fixed value in the steady-state method of ^15^O-labeled gases [9, 21], and therefore, regional differences in distribution volume of ^15^O-labeled water can affect measurement of CBF, OEF and CMRO_2_ by PET with ^15^O-labeled gases [13]. To estimate systemic errors in measurement of OEF caused by regional differences in distribution volume of ^15^O-labeled water, a simulation study was performed. The tissue radioactivities for brain regions of ^15^O_2_ and C^15^O_2_ during the steady state were generated according to the steady-state method with CBF values between 0.1 and 0.9 mL/mL/min in five steps and with distribution volume values between 0.7 and 1.3 mL/mL in seven steps. OEF values were assumed to be 0.4 and 0.6. CBV was assumed to be 0.05 mL/mL. From these tissue radioactivities, CBF and OEF values were calculated by the steady-state method with an assumed distribution volume of 1.0 mL/mL [9, 21]. Calculated CBF and OEF values were compared with the assumed CBF and OEF values.

#### Errors in measurement of OEF caused by a mixture of gray and white matter

The limited spatial resolution of the PET scanner causes a mixture of gray and white matter in voxels. This mixture causes errors in the calculation of CBF [29–31]. To estimate systemic errors in measurements of OEF caused by a mixture of gray and white matter, a simulation study was performed. The tissue radioactivities for brain regions of ^15^O_2_ and C^15^O_2_ during the steady state were generated as mixtures of gray and white matter according to the steady-state method. The CBF values for the gray and white matter were assumed to be 0.8 and 0.2 mL/mL/min, respectively. The distribution volumes of the ^15^O-labeled water for gray and white matter were both assumed to be 1.0 mL/mL. The OEF values for gray and white matter were both assumed to be 0.4. CBV values for the gray and white matter were assumed to be 0.05 and 0.02 mL/mL, respectively. The difference between true CBF, calculated as CBF = 0.8 mL/100 mL/min × gray matter fraction + 0.2 mL/mL/min × white matter fraction, and CBF calculated by the steady-state method on the basis of the generated heterogeneous tissue radioactivity was estimated, where the fraction of gray matter per given ROI varied from 0 to 1.0. The difference between assumed OEF and OEF calculated by the steady-state method on the basis of the generated heterogeneous tissue radioactivity was also estimated, where the fraction of gray matter per given ROI varied from 0 to 1.0.

## Results

**Fig. 1:**
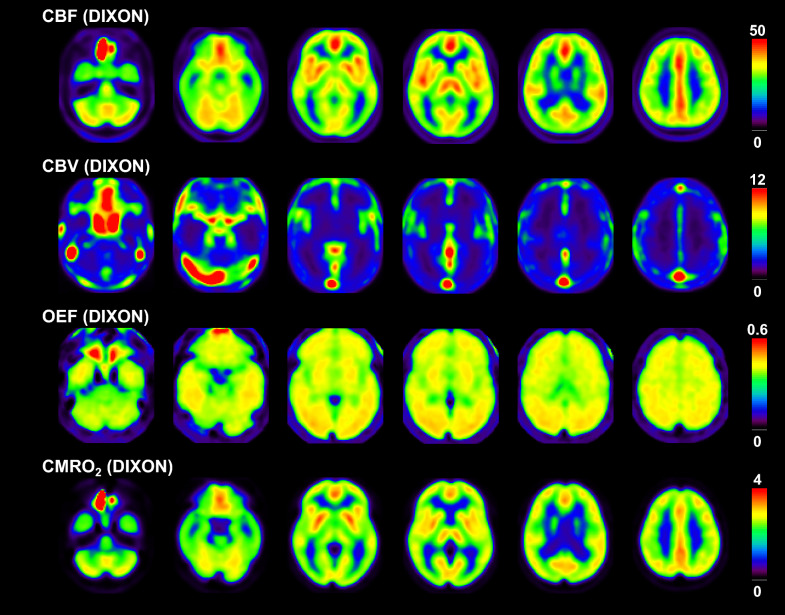
Averaged anatomically standardized PET images with attenuation correction by DIXON. Scale maximum values are 50 mL/100 mL/min, 12 mL/100 mL, 0.6, and 4 mL/100 mL/min for CBF, CBV, OEF, and CMRO_2_ images, respectively. The image slices are the same as Fig. 3

**Fig. 2:**
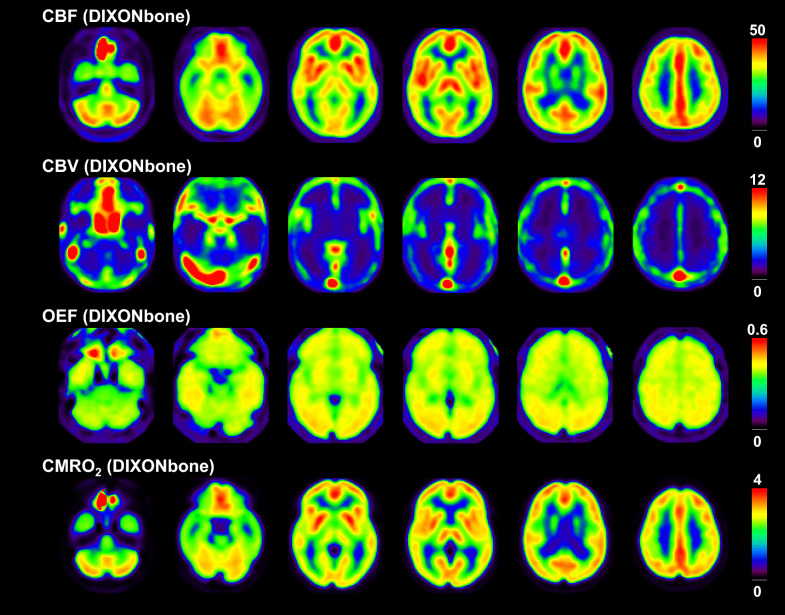
Averaged anatomically standardized PET images with attenuation correction by DIXONbone. Scale maximum values are 50 mL/100 mL/min, 12 mL/100 mL, 0.6, and 4 mL/100 mL/min for CBF, CBV, OEF, and CMRO_2_ images, respectively. The image slices are the same as Fig. 3

The averaged anatomically standardized PET images with attenuation correction by DIXON and DIXONbone are shown in Figs. 1 and 2, respectively. Tables 1 and 2 give mean and SD values of CBF, CBV, OEF, and CMRO_2_ with attenuation correction by DIXON and DIXONbone, respectively. The OEF value in the pons was significantly smaller than those in the other brain regions without the cerebellar cortex. The OEF value in the parahippocampal gyrus was significantly smaller than those in the other cerebral cortical regions. The CMRO_2_ value in the parahippocampal gyrus was also significantly smaller than those in the other cerebral cortical regions. The OEF value in the lateral side of the occipital cortex was largest among the cerebral cortical regions.

**Table 1:** Values of CBF, CBV, OEF and CMRO_2_ with attenuation correction by DIXON for each brain region

Region	CBF	CBV	OEF	CMRO_2_
Cerebellum	32.7 ± 3.8	2.5 ± 0.3	0.36 ± 0.07	2.4 ± 0.4
Pons	27.2 ± 6.1	3.5 ± 0.5	0.28 ± 0.08	1.5 ± 0.3^(1)^
Thalamus	39.6 ± 4.8^(1,2)^	2.7 ± 0.5	0.36 ± 0.06^(2)^	2.8 ± 0.4^(2)^
Putamen	40.4 ± 7.0^(1,2)^	2.0 ± 0.2^(2)^	0.41 ± 0.07^(2,3)^	3.3 ± 0.5^(1,2,3)^
Frontal base	29.5 ± 4.4^(3,4)^	2.2 ± 0.2^(2)^	0.40 ± 0.07^(2,3)^	2.3 ± 0.2^(2,3,4)^
Frontal convexity	31.8 ± 4.8^(3,4)^	2.6 ± 0.4	0.39 ± 0.07^(2)^	2.5 ± 0.4^(2,4)^
Parahippocampal gyrus	28.7 ± 3.8^(1,3,4)^	3.4 ± 0.6^(1,3,4,5)^	0.33 ± 0.08^(2,4,5,6)^	1.9 ± 0.3^(2,3,4,5,6)^
Lateral temporal cortex	28.3 ± 5.2^(3,4)^	3.2 ± 0.5^(4,5)^	0.38 ± 0.07^(2,7)^	2.2 ± 0.4^(2,3,4)^
Occipital cuneus	30.2 ± 4.2^(3,4)^	3.4 ± 0.4^(1,3,4,5,6)^	0.40 ± 0.06^(2,3,7)^	2.4 ± 0.3^(2,4,7)^
Occipital lateral	23.2 ± 2.9^(1,3,4,6,9)^	2.4 ± 0.3^(2,7,8,9)^	0.44 ± 0.06^(1,2,3,7,8)^	2.1 ± 0.3^(2,3,4,6)^
Inferior parietal lobule	30.5 ± 3.6^(3,4,10)^	2.8 ± 0.5^(4)^	0.40 ± 0.06^(2,7)^	2.5 ± 0.4^(2,4,7,8,10)^
Centrum semiovale	12.8 ± 1.4^(1–11)^	1.2 ± 0.2^(1–11)^	0.37 ± 0.07^(2)^	0.9 ± 0.2^(1–11)^

Values are shown as mean ± SD. Units are mL/100 mL/min, mL/100 mL, and mL/100 mL/min for CBF, CBV, and CMRO_2_, respectively. Significant differences between from (1) cerebellum, (2) pons, (3) thalamus, (4) putamen, (5) frontal base, (6) frontal convexity, (7) Parahippocampal gyrus, (8) Lateral temporal cortex, (9) Occipital cuneus, (10) Occipital lateral, (11) Inferior parietal lobule: *P* < 0.001 (paired *t* test, *P* < 0.05 adjusted for multiple comparisons)

**Table 2:** Values of CBF, CBV, OEF and CMRO_2_ with attenuation correction by DIXONbone for each brain region

Region	CBF	CBV	OEF	CMRO_2_
Cerebellum	40.1 ± 4.9	2.5 ± 0.2	0.37 ± 0.08	3.0 ± 0.4
Pons	30.7 ± 7.0^(1)^	3.7 ± 0.5	0.28 ± 0.08	1.7 ± 0.3^(1)^
Thalamus	44.4 ± 5.4^(2)^	2.8 ± 0.5	0.36 ± 0.07^(2)^	3.2 ± 0.4^(2)^
Putamen	46.4 ± 7.6^(2)^	2.1 ± 0.2^(2)^	0.42 ± 0.08^(2,3)^	3.8 ± 0.4^(2,3)^
Frontal base	35.3 ± 6.1^(3,4)^	2.2 ± 0.3^(2)^	0.40 ± 0.07^(2)^	2.8 ± 0.3^(2,4)^
Frontal convexity	39.0 ± 5.8	2.6 ± 0.4	0.39 ± 0.07^(2)^	3.0 ± 0.3^(2)^
Parahippocampal gyrus	31.3 ± 4.2^(1,3,4)^	3.5 ± 0.7	0.33 ± 0.08^(2,4,5)^	2.1 ± 0.3^(1,3,4,5,6)^
Lateral temporal cortex	33.1 ± 5.9^(3,4)^	3.3 ± 0.5	0.39 ± 0.07^(2)^	2.6 ± 0.3^(2,3,4)^
Occipital cuneus	33.7 ± 5.2^(3,4)^	3.6 ± 0.4^(1,4,5)^	0.41 ± 0.07^(2,3,7)^	2.7 ± 0.3^(2,4,7)^
Occipital lateral	27.1 ± 3.5^(1,3,4)^	2.5 ± 0.4^(9)^	0.45 ± 0.06^(2,3,7)^	2.4 ± 0.4^(3,4,6)^
Inferior parietal lobule	36.0 ± 3.6^(7)^	2.9 ± 0.6	0.41 ± 0.06^(2)^	3.0 ± 0.3^(2,4,7,10)^
Centrum semiovale	13.2 ± 1.3^(1–11)^	1.2 ± 0.2^(1–11)^	0.37 ± 0.08^(2)^	1.0 ± 0.2^(1–11)^

Values are shown as mean ± SD. Units are mL/100 mL/min, mL/100 mL, and mL/100 mL/min for CBF, CBV, and CMRO_2_, respectively. Significant differences between from (1) cerebellum, (2) pons, (3) thalamus, (4) putamen, (5) frontal base, (6) frontal convexity, (7) Parahippocampal gyrus, (8) Lateral temporal cortex, (9) Occipital cuneus, (10) Occipital lateral, (11) Inferior parietal lobule: *P* < 0.001 (paired *t* test, *P* < 0.05 adjusted for multiple comparisons)

**Fig. 3:**
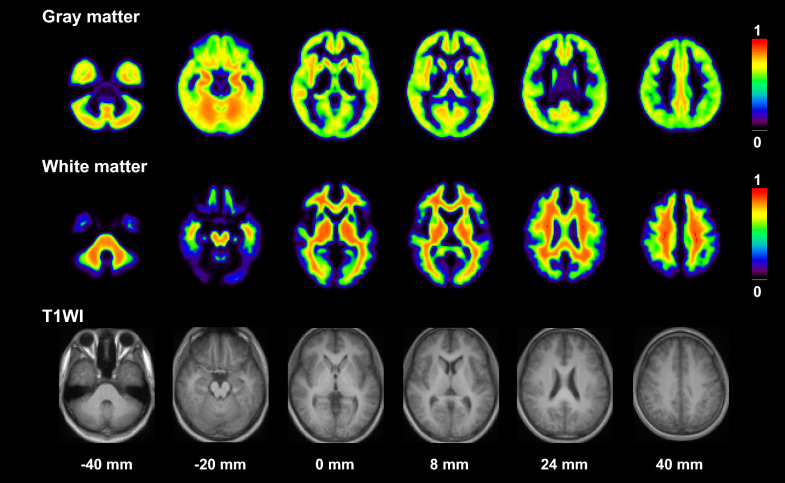
Averaged gray and white matter images extracted from anatomically standardized MR images. All images are transaxial sections parallel to the anterior–posterior commissure (AC–PC) line. The slice positions are − 40, − 20, 0, 8, 24 and 40 mm from the AC–PC line

The averaged gray and white matter images extracted from anatomically standardized MR images are shown in Fig. 3. The gray and white matter fractions for each ROI are given in Table 3. The highest gray matter fraction was observed in the parahippocampal gyrus.

**Table 3:** Values of gray and white matter fraction for each brain region

	Fraction	
Region	Gray matter	White matter
Cerebellum	0.78 ± 0.04	0.18 ± 0.05
Pons	0.06 ± 0.01^(1)^	0.93 ± 0.01^(1)^
Thalamus	0.41 ± 0.07^(1,2)^	0.59 ± 0.07^(1,2)^
Putamen	0.59 ± 0.08^(1,2)^	0.41 ± 0.08^(1,2)^
Frontal base	0.62 ± 0.03^(1,2,3)^	0.27 ± 0.06^(2,3)^
Frontal convexity	0.62 ± 0.05^(1,2,3)^	0.20 ± 0.06^(2,3,4)^
Parahippocampal gyrus	0.92 ± 0.02^(1,2,3,4,5,6)^	0.03 ± 0.01^(1,2,3,4,5,6)^
Lateral temporal cortex	0.65 ± 0.05^(2,3,7)^	0.24 ± 0.08^(2,3,4,7)^
Occipital cuneus	0.57 ± 0.08^(1,2,3,7)^	0.37 ± 0.09^(1,2,3,7) (8)^
Occipital lateral	0.51 ± 0.04^(1,2,3,5,7,8)^	0.45 ± 0.06^(1,2,3,5,6,7,8)^
Inferior parietal lobule	0.62 ± 0.04^(1,2,3,7,10)^	0.25 ± 0.09^(2,3,7,10)^
Centrum semiovale	0.01 ± 0.01^(1–11)^	0.99 ± 0.01^(1–11)^

Values are shown as mean ± SD. Unit is mL/mL. Significant differences between from (1) cerebellum, (2) pons, (3) thalamus, (4) putamen, (5) frontal base, (6) frontal convexity, (7) Parahippocampal gyrus, (8) Lateral temporal cortex, (9) Occipital cuneus, (10) Occipital lateral, (11) Inferior parietal lobule: *P* < 0.001 (paired *t* test, *P* < 0.05 adjusted for multiple comparisons)

**Fig. 4:**
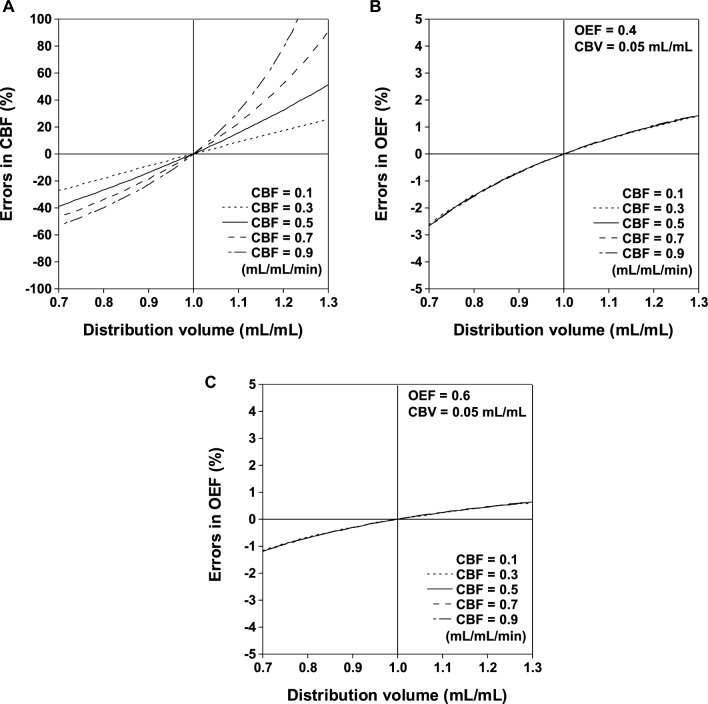
Systemic errors in measurement of CBF (**A**) and OEF (**B**, **C**) caused by regional differences in distribution volume of ^15^O-labeled water

Systemic errors in the measurement of CBF and OEF caused by regional differences in the distribution volume of ^15^O-labeled water are shown in Fig. 4. The differences in the distribution volume of ± 0.1 mL/mL yielded CBF errors within ± 20% when CBF value was 0.5 mL/mL/min. The differences in the distribution volume of ± 0.1 mL/mL yielded OEF errors within ± 1% when CBF values were 0.1 to 0.9 mL/mL/min.

**Fig. 5:**
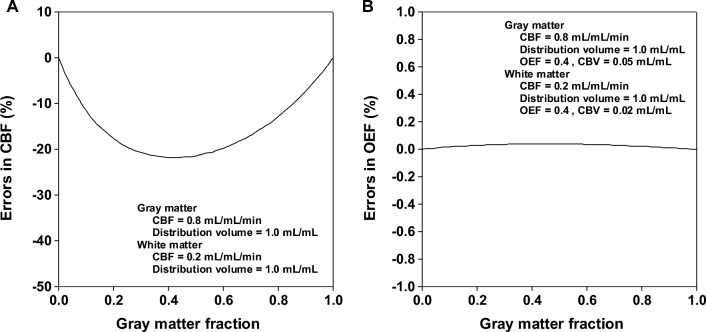
Systemic errors in measurement of CBF (**A**) and OEF (**B**) caused by a mixture of gray and white matter

The systemic errors in measurement of CBF and OEF caused by a mixture of gray and white matter are shown in Fig. 5. CBF was systematically underestimated because of such mixing. When the gray matter fraction was 0.1–0.9, the underestimation of CBF was around 10–20%. The errors in OEF were around 0% for each gray matter fraction.

## Discussion

The regional differences in OEF values in the brain were observed in the present study. There are some sources of systemic errors in the PET measurement of CBF, OEF, and CMRO_2_, i.e., regional differences in radiotracer appearance time in the brain, regional differences in distribution volume of ^15^O-labeled water, and regional differences in a mixture of gray and white matter [13]. In the steady-state method, since ^15^O_2_ and C^15^O_2_ gases were continuously administrated during PET scanning [9, 21], regional differences in radiotracer appearance time in the brain may not be considered.

Regional differences in the distribution volume of ^15^O-labeled water can affect measurement of CBF, OEF and CMRO_2_ by PET with ^15^O-labeled water or gas [13]. In the simulation studies of the present report, differences in distribution volume of ± 0.1 mL/mL yielded OEF errors within ± 1%, whereas differences in distribution volume of ± 0.1 mL/mL yielded CBF errors within ± 20% almost same as previous report [29]. In addition, almost identical distribution volumes among the cerebral cortical regions and thalamus have been reported [32, 33]. Thus, errors in OEF caused by regional differences in the distribution volume of ^15^O-labeled water must be assumed to be negligible.

The mixture of gray and white matter in the ROIs causes errors in the calculation of CBF [29–31]. In the simulation studies of the present report, CBF was systematically underestimated because of such mixing; however, errors in OEF were around 0%. It has been reported that errors in measurement of OEF caused by a mixture of gray and white matter was less than 1% in the steady-state method, while the maximum underestimation of 18% was observed in CBF [17, 29]. Thus, the effects of tissue heterogeneity must be negligible on measurement of OEF by the steady-state method.

The OEF value in the pons was smaller than those in the other brain regions same as previous reports [8, 10, 11, 16]. This indicates that excessive blood flow is supplied as compared with oxygen demand in this region which plays a role of important functions for respiratory and circulation. In addition, a large capacity for vasodilatation during hypercapnia and a large capacity for vasoconstriction during hypocapnia have been shown in the pons, indicating marked vascular responsiveness [14].

The OEF value in the parahippocampal gyrus was smaller than those in the other cerebral cortical regions same as previous reports [8, 10, 11, 16]. This indicates excessive blood flow are supplied as compared with oxygen demand in this region which is of central interest in the complex pathophysiology of Alzheimer’s disease. The highest gray matter fraction was observed in the parahippocampal gyrus same as previous reports [34]. However, CBF was not greater in this region than in the other regions. Thus, the parahippocampal gyrus has been reported to show the lowest blood flow values per gray matter fraction [34]. In this study, CMRO_2_ value in the parahippocampal gyrus was also significantly smaller than those in the other cerebral cortical regions, similar to previous reports [10, 11, 16]. Thus, CMRO_2_ value per gray matter fraction in this region must be lowest among the cerebral cortical regions.

The OEF value in the lateral side of the occipital cortex was largest among the cerebral cortical regions, similar to previous reports [8, 10, 15, 16]. It has been reported that significant relative hypoperfusion during hypercapnia and significant relative hypoperfusion during hypocapnia were observed in the temporal, temporo-occipital, and occipital cortices, indicating that cerebral vascular tone at rest might incline toward vasodilatation [14]. It has also been reported that the vascular mean transit time, i.e., CBV/CBF was longest in the temporooccipital cortex in brain, which might relate to that cerebral vascular tone at rest might incline toward vasodilatation [13]. A proportional relationship between capillary transit time and OEF has been reported [35]. It has also been reported that the long transit time of red blood cell in capillaries might allow the transport of sufficient oxygen [36]. Thus, the longest vascular mean transit time might contribute to the largest OEF value in the lateral side of the occipital cortex.

**Fig. 6:**
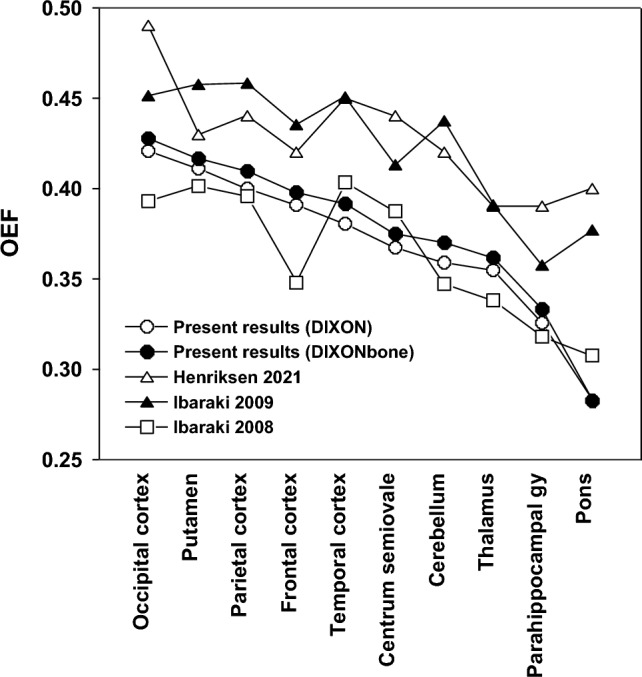
Regional distribution of OEF in the brain as compared with previous reports [11, 16, 37]. The values of the cuneus and lateral side of the occipital cortex are averaged as the occipital cortex. The values of the base and convexity side of the frontal cortex are averaged as the frontal cortex. The values in the brainstem and the mesial temporal in Henriksen’s report are plotted as the pons and parahippocampal gyrus, respectively [16]

The regional distribution of OEF in the brain as compared with previous reports are shown in Fig. 6 [11, 16, 37]. Although there are limitations of small sample size in the present study, similar regional distribution of OEF in brain were observed between the present results and those of the previous reports.

The similar regional differences in OEF in the brain were observed between with attenuation corrections by DIXON and DIXONbone. It has been reported that the OEF values that were relative values corresponding to ratios of CMRO_2_ to CBF were not affected by the attenuation correction methods, while the CBF, CBV, and CMRO_2_ values that included scale factors to radioactivity concentration in the brain might be affected by the attenuation correction methods [12]. Thus, effects of attenuation correction methods on regional differences in OEF in brain might not be observed.

The regional distribution of the cerebral metabolic rate of glucose measured by PET with [^18^F]fluorodeoxyglucose ([^18^F]FDG) in man was investigated, and these reports showed the lowest cerebral glucose metabolism in the cerebellum as compared with the cerebral neocortical regions [38–40]. In the present study, the CMRO_2_ in the cerebellum was larger than those in the cerebral cortical regions same as previous reports [8, 10, 11]. Since cerebral glucose metabolism is mainly aerobic metabolism [41], this discrepancy between CMRO_2_ and cerebral metabolic rate of glucose in the cerebellum might be able to be explained by a lower lumped constant, that is, the ratio of influx rate constants across the blood–brain barrier for fluorodeoxyglucose to glucose [42, 43], in this region.

## Conclusions

The regional differences in OEF in the brain were investigated in detail, with consideration of systemic errors in PET measurement. The OEF value in the pons was significantly smaller than those in the other brain regions. The OEF value in the parahippocampal gyrus was significantly smaller than those in the other cerebral cortical regions. The OEF value in the lateral side of the occipital cortex was largest among the cerebral cortical regions. Simulation studies revealed that errors in OEF caused by regional differences in distribution volume of ^15^O-labeled water and errors in OEF caused by a mixture of gray and white matter must be negligible.

## Data Availability

The data sets used and/or analyzed during the current study are available from the corresponding author on reasonable request.
